# Design of Rigidity and Breaking Strain for a Kirigami Structure with Non-Uniform Deformed Regions

**DOI:** 10.3390/mi10060395

**Published:** 2019-06-14

**Authors:** Hiroki Taniyama, Eiji Iwase

**Affiliations:** Department of Applied Mechanics, Waseda University, 3-4-1 Okubo, Shinjuku-ku, Tokyo 169-8555, Japan; taniyama@iwaselab.amech.waseda.ac.jp

**Keywords:** flexible device, stretchable electronic substrate, kirigami structure, mechanical metamaterials

## Abstract

We modeled a kirigami structure by considering the influence of non-uniform deforming cuts in order to theoretically design the mechanical characteristics of the structure. It is known that the end regions of kirigami structures are non-uniformly deformed when stretched, because the deformation is inhibited at the regions close to both the ends connected to the uncut region in the longitudinal direction. The non-uniform deformation affects the overall mechanical characteristics of the structure. Our model was intended to elucidate how cuts at both ends influence these characteristics. We focused on the difference in the deformation degree caused by a cut between the regions close to the ends and the center of the stretched kirigami device. We proposed a model comprising of connected springs in series with different rigidities in the regions close to the ends and the center. The spring model showed good prediction tendency with regard to the curve of the stress–strain diagram obtained using the tensile test with a test piece. Therefore, the results show that it is possible to theoretically design the mechanical characteristics of a kirigami structure, and that such a design can well predict the influence of cuts, which induce non-uniform deformation at both ends.

## 1. Introduction

Our objective in this study was to design the mechanical characteristics—such as rigidity and breaking point—of a kirigami structure theoretically by means of a model that considers the influence of cuts, which induce non-uniform deformation at both ends of the stretched kirigami structure.

In recent years, many researchers have conducted investigations using sheets with incised periodic cuts, or so-called kirigami structures. Such structures show interesting mechanical characteristics [1,2,3,4,5,6,7,8,9,10,11,12,13,14,15,16,17,18,19,20,21,22,23,24,25,26,27]. For example, a kirigami structure can tune the rigidity and breaking strain of the overall device by virtue of changes in its length or the density of cuts in the structure [1,2,3,4,5,6,7,8,9]. Shyu et al. elucidated the tendency of the stress–strain diagram by stretching the kirigami structure using different parametric conditions and by analyzing the deformations of the structure with finite element modelling [1]. Isobe and Okumura studied the relation between the geometric parameters and rigidity of a kirigami-structured device using the balance of elastic strain energies. They considered a beam model of the kirigami structure and fixed the number of cuts per unit cycle in the longitudinal direction of the structure [2]. Hwang and Bartlett designed a kirigami structure to specifically increase the breaking strain by approximately 1.7 times. The structure was novel in that “minor cuts” were also introduced at both cut ends [3]. Wang et al. developed a kirigami-patterned stretchable conductive film (KSCF) fabricated by laser cutting composites of carbon nanotube conductive networks and an elastic polydimethylsiloxane substrate. Interestingly, the normalized resistance change of the KSCF was 0.10, even after 5000 stretching tests using 0 to 400% strain [4]. Tang and Yin controlled the inclining direction of out-of-plane deformation in a kirigami structure by devising patterns of notches at the front and reverse sides [5]. Lei and Nakatani analyzed the transiting process from in-plane to out-of-plane deformation by modeling a kirigami structure with a beam combination [6]. Rafsanjani and Bertoldi [7] and Tang and Yin [8] designed rigid sheet kirigami structures with square cut units. Moshe et al. proposed an elastic change framework to understand kirigami mechanics in thin sheets with perforations [9]. Dias et al. designed linear actuators that can perform four fundamental forms of linear actuation, that is, roll, pitch, yaw, and lift, by tuning the locations and arrangements of the cuts [10]. Various practical applications have been suggested by changing the mechanical characteristics of kirigami structures, such as increasing the breaking strain of the material and testing steric out-of-plane deformations by stretching the structure [11,12,13,14,15,16,17,18,19,20,21,22,23,24,25,26]. Such applications include strain sensors [11,12,13,14], stretchable heaters [15], solar cells with solar tracking systems [16], bioprobes [17], crawling robots [18], artificial muscles [19], soft deployable reflectors [20], self-folding hinges [21,22], metamaterial bricks [23], and adhesives with tunable anisotropic adhesive strength [24]. It has been confirmed that a kirigami structure becomes non-uniformly deformed when it is stretched, because the deformation is inhibited at regions close to both ends connected to the uncut region in the longitudinal direction [1,2,3,4,5,11,12,13,14,15,16,17]. The non-uniform deforming affects the mechanical characteristics of the overall device. However, the influence of non-uniform deformation on these characteristics has not been analyzed and designed theoretically. In a conventional kirigami structure, the issue of the non-uniform deforming affects has been ignored by increasing the number of patterns per unit cycle of the kirigami structure. This conventional approach can reduce the influence of non-uniform deformation but is not applicable when the range including a kirigami structure is determined or when it is desired to have as low a range as possible. For example, consider a kirigami structure used in an electronic circuit to obtain stretchable wiring parts; it is important to reduce the number of wiring parts to the greatest possible extent and increase the area of the substrate region available for mounting electronic elements. If the mechanical characteristics of the kirigami structure can be theoretically designed while considering non-uniform deformation, it is possible to realize the required mechanical characteristics by making cuts in the minimum range.

In this study, we modeled a kirigami structure to elucidate how cuts at both ends, which induce non-uniform deforming, influence the mechanical characteristics of the overall device. We focused on the difference in the degree of deformation caused by cuts between regions close to the ends and the center of a stretched kirigami device. We proposed a model of connected springs in series based on the hypothesis that there is different rigidity of the regions close to the ends and the center. We derived formulas from the connected springs model, showing the relation between the number of patterns per unit cycle and the mechanical characteristics of the overall device containing the kirigami structure. Comparing the derived model formula and the value measured using the tensile test, we examined whether the proposed model is applicable to kirigami structures. The test piece with a kirigami structure was used for the tensile test. It was made of polyimide (PI) copper (Cu) substrate, which is often used as a film substrate in a flexible device. Section 2 describes the definition of a kirigami structure, and the theory of the spring model proposed in this study and tensile test method. Section 3 describes the mechanical characteristics of a kirigami structure by tensile test. Moreover, we examined whether the proposed spring model was applicable to the kirigami structure by comparing the model values to the spring model values measured by the tensile test. Section 4 describes the conclusions of this study.

## 2. Theory and Methods

### 2.1. Definition of a Kirigami Structure

**Figure 1:**
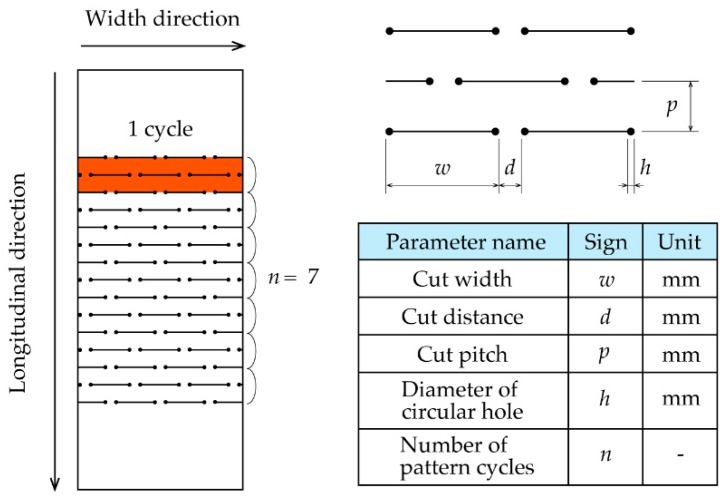
Parameters of a kirigami structure and cut patterns.

In this paper, we define a kirigami structure as shown in Figure 1. The kirigami structure has periodic cuts in the width direction, and the set of cuts lined in the width direction is repeated in the longitudinal direction. Here, two repetitive cut lines are defined as one pattern cycle. A circular hole is provided at each end of the cut to reduce stress concentration because it is known that when a kirigami structure is stretched, stress is concentrated at both ends of the cut, which could induce cracks [27]. The various dimension parameters that can describe a kirigami structure include cut width *w* [mm], cut distance *d* [mm], cut pitch *p* [mm], diameter of circular hole *h* [mm], and number of pattern cycles *n*. The mechanical characteristics of a kirigami structure or a device that includes this structure can change with any modifications to the dimension parameters [1,2,3,4,5,6,7,8,9,10]. The kirigami structure shown in Figure 1 is deformed in the three-dimensional and out-of-plane directions after in-plane deformation in two dimensions.

### 2.2. Theory of the Spring Model Proposed in This Study

**Figure 2:**
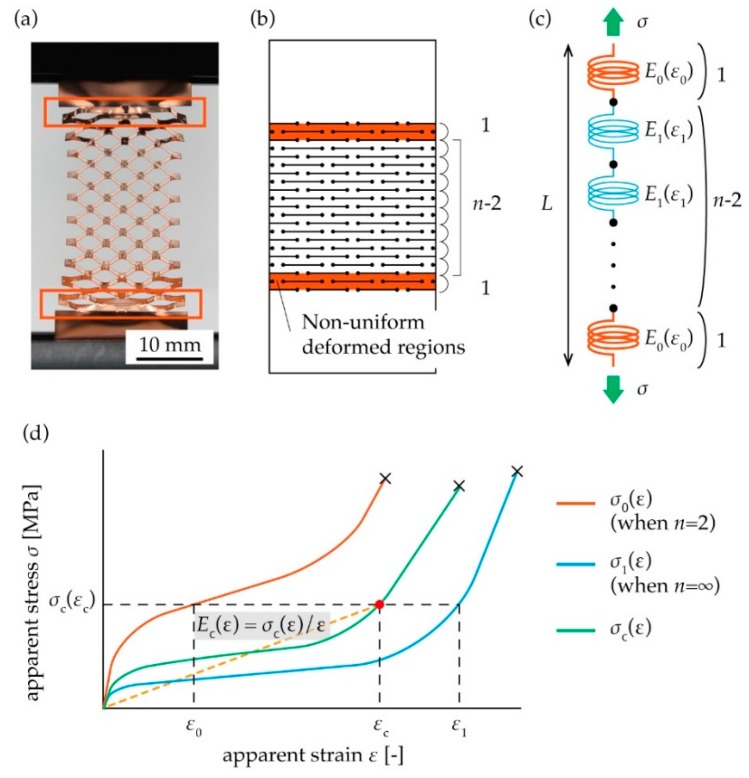
(**a**) Photograph of a stretched kirigami structure. (**b**) Definition of non-uniform deformed regions at both ends. (**c**) A spring model of the kirigami structure, in which two hard springs with an elongation stress per strain *E*_0_(*ε*_0_) sandwich *n* − 2 soft springs with an elongation stress per strain *E*_1_(*ε*_1_). (**d**) The combined stress–strain curve of the whole spring *σ*_c_(*ε*) can be obtained from stress–strain curves *σ*_0_(*ε*) and *σ*_1_(*ε*) of each spring.

The stretching of the kirigami structure shown in Figure 1 results in the shape seen in Figure 2a. As shown in Figure 2a, there are differences in deformation between the regions in the center and non-uniformly deformed regions. The deformation in one pattern cycle at the edge is non-uniform compared with the other. Based on this fact, we divided a kirigami structure into three regions, with two regions showing non-uniform deformation at both ends, and one region showing uniform deformation at the center, as shown in Figure 2b. The three divided regions are considered as a model of springs in series, in which two hard springs sandwich *n* − 2 soft springs, as shown in Figure 2c. A typical stress–strain diagram of a kirigami structure shows non-linear curves, as shown in Figure 2d. Thus, we considered elongation stress per strain *E*(*ε*) and breaking strain *ε*_cb_ to characterize a kirigami structure mechanically. The elongation stress per strain *E*(*ε*) is a physical value that extends Young’s modulus beyond the linear region. Therefore, if the structure is in the linear region, *E*(*ε*) is constant and takes the same value as Young’s modulus. The definition of the elongation stress per strain is depicted as seen in Equation (1) and Figure 2d.

(1) $$E\left(\varepsilon \right)\overset{\mathrm{def}}{=}\frac{\sigma \left(\varepsilon \right)}{\varepsilon }$$

Then, we considered that a combined spring, namely the two hard springs and the *n* − 2 soft springs connected in series, are stretched by stress *σ*, as shown in Figure 2c. We assumed that the strains of the two hard springs and the *n* − 2 soft springs are *ε*_0_ and *ε*_1_, respectively, and the combined strain of the whole spring of length *L* is *ε*_c_. Considering the displacement by stress *σ*_c_, we obtained Equation (2).

(2) $$\varepsilon_{c}L=\frac{2}{n}\varepsilon_{0}L+\frac{n-2}{n}\varepsilon_{1}L$$

In Equation (2), we assumed that each spring has the same length (*L*/*n*), corresponding to the cut pitch of the kirigami structure. Then, we posited that the two hard springs and the *n* − 2 soft springs have an elongation stress per strain *E*_0_(*ε*_0_) and *E*_1_(*ε*_1_), respectively, as shown in Figure 2c. Considering the balance of stress when the springs connected in series are stretched by stress *σ*, the combined elongation stress per strain of the whole spring *E*_c_(*ε*_c_) is represented as

(3) $$\varepsilon_{c}E_{c}\left(\varepsilon_{c}\right)=\varepsilon_{0}E_{0}\left(\varepsilon_{0}\right)=\varepsilon_{1}E_{1}\left(\varepsilon_{1}\right)$$

In Equation (3), we assumed that all the springs are of the same cross-sectional area, corresponding to the cross-sectional area of the substrate. From Equations (2) and (3), the elongation stresses per strain of each spring *E*_0_(*ε*_0_) and *E*_1_(*ε*_1_) are used to express the elongation stress per strain of the whole spring *E*_c_(*ε*_c_), as shown in Equation (4) and Figure 2d.

(4) $$E_{c}\left(\varepsilon_{c}\right)=\frac{nE_{0}\left(\varepsilon_{0}\right)E_{1}\left(\varepsilon_{1}\right)}{2E_{1}\left(\varepsilon_{1}\right)+\left(n-2\right)E_{0}\left(\varepsilon_{0}\right)}$$

We used the model of Equation (4) to analyze the kirigami in Figure 1. In our analysis, if we obtained *E*_0_(*ε*_0_) and *E*_1_(*ε*_1_) experimentally, we could obtain the elongation stress per strain *E*_c_(*ε*_c_) for any number of pattern cycles *n*. The values *E*_0_(*ε*_0_) and *E*_1_(*ε*_1_) were obtained by fitting to the model of Equation (4) using the measured value *E*_c_(*ε*_c_) on various number of pattern cycles *n*. In addition, we considered the connected springs in series model to elucidate the relationship between *n* and breaking strain. The breaking strains of the springs with the respective elongation stress per strain *E*_0_(*ε*_0_) and *E*_1_(*ε*_1_) are defined as *ε*_0b_ and *ε*_1b_ respectively. If we assume the breaking strains *ε*_0b_ and *ε*_1b_ are almost the same, we can obtain the combined breaking strain *ε*_cb_ of the whole spring is as follows from Equation (2).

(5) $$\varepsilon_{\mathrm{cb}}=\frac{2}{n}\varepsilon_{0b}+\frac{n-2}{n}\varepsilon_{1b}$$

In our analysis, the values *ε*_0b_ and *ε*_1b_ were obtained by fitting to the model of Equation (5) using the measured values $\varepsilon_{\mathrm{cb}}$ on various number of pattern cycles *n*. 

### 2.3. Tensile Testing Method

**Figure 3:**
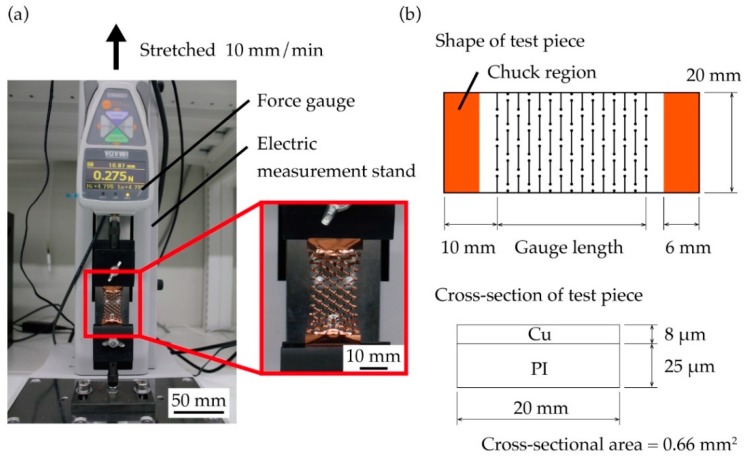
(**a**) Photograph of the tensile test setup, and (**b**) definition of the test piece shape in the tensile test and parameter dimensions.

Comparing the derived formula and results from the tensile test, we examined the applicability of the proposed model to the kirigami structure. Test pieces for the tensile test were fabricated using PI-Cu substrate (Toray Advanced Materials Korea, Metaroiyal^®^), and a film of Cu was formed by sputtering and electrolytic Cu plating. The thicknesses of the PI and Cu layers on the substrate were 25 µm and 8 µm, respectively. The test piece for the tensile test was shaped as a bar and the kirigami structure was included in the central region. The test piece included non-cut areas, which measured 10 mm in the longitudinal direction, at both ends. The kirigami structure for the test piece was cut out using an ultraviolet laser beam machine (Osada Photonics International, OLMUV-355-5A-K) with a wavelength of 355 nm. The tensile test was performed by stretching the test piece using the tensile testing machine with an integrated force gauge (IMADA, ZTA-5N) and electric measurement stand (IMADA, MX2-500N). The test piece was fixated by jigs (IMADA, FC-41U, FC-41U-F) equipped with urethane rubber on one side, so as not to break the chuck region on stretching. Figure 3 shows the experimental setup for the tensile test. The test piece was fixed by the jig so that the tested length was 6 mm from both ends in the longitudinal direction. The static tensile test was selected in order to evaluate the mechanical characteristics of the kirigami structure, and the lifting speed of the tensile test was 10 mm/min, which was sufficiently slow compared to the length of the test piece. When stretching the test piece with the tensile testing machine, load and elongation were monitored by the force gauge, and the load–elongation diagram was prepared for the kirigami structure. The test piece started stretching from its bended state and, once the load reached 0.01 N, the zero position of load–elongation diagram was set (namely, the load and elongation were zero at this point). The stress–strain diagram was produced using the obtained load–elongation diagram. The obtained load was divided by the cross-sectional area of the substrate, and the strain was divided by the gauge length of the test piece. Here, we considered cross-sectional area of the substrate without cuts and defined length between the ends of the cuts in the kirigami structure as the gauge length of the test piece. Figure 3b shows definition of the cross-sectional area and gauge length of the test piece. The dimension parameters of the test piece were fixed as follows: cut width *w*, cut distance *d*, cut pitch *p*, diameter of each round hole *h*, and thickness of the substrate were 5 mm, 1 mm, 1 mm, 0.3 mm, and 33 µm, respectively. Six types of pattern cycles (*n*) were used (3, 5, 10, 15, 20, and 25). A tensile test was conducted five times for each of the six patterns.

## 3. Results and Discussion

**Figure 4:**
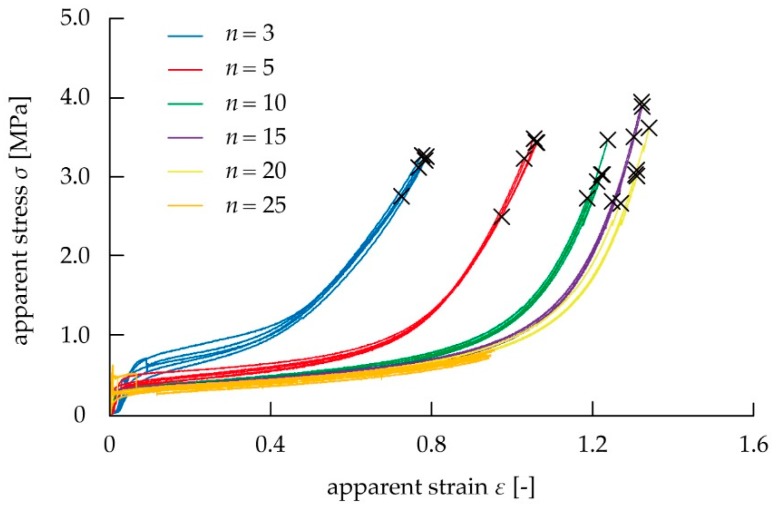
Stress–strain diagram when stretching the kirigami structure for different values of *n*.

**Figure 5:**
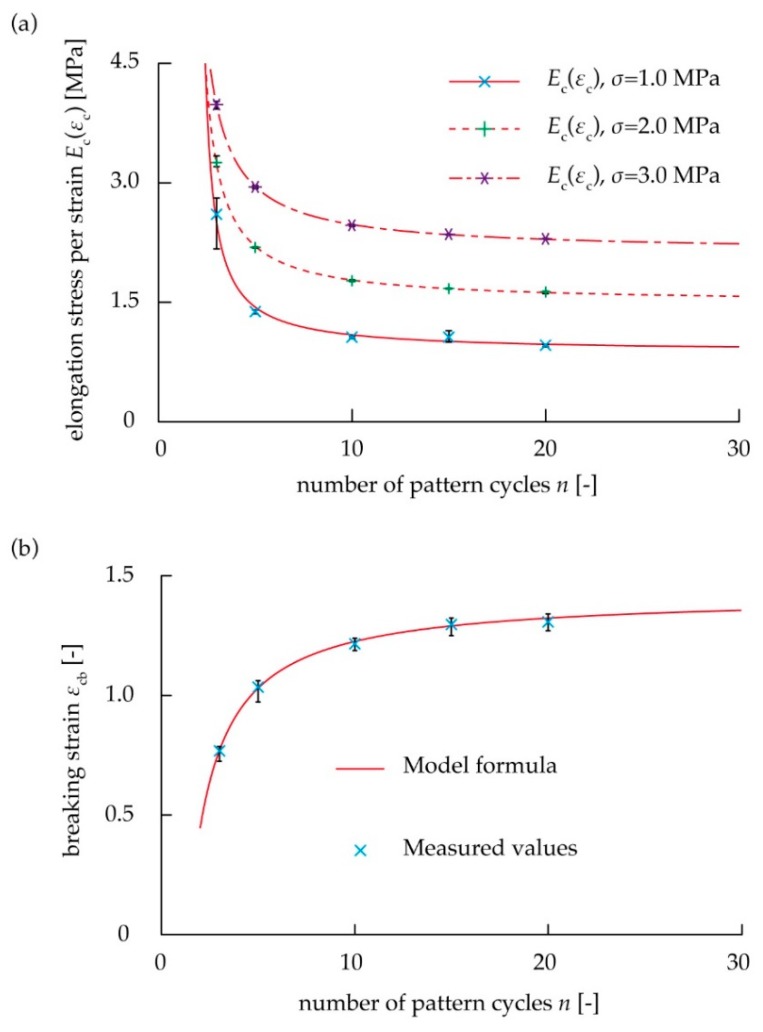
Comparison between fitting curve by theoretical and measured values: (**a**) Relation between *n* and elongation stress per strain, (**b**) Relationship between *n* and breaking strain.

**Figure 6:**
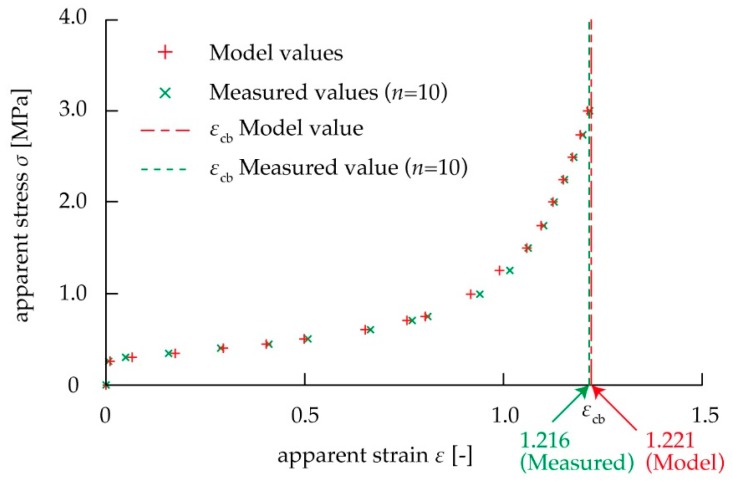
Comparison of values calculated using the spring model and values in the stress–strain diagram from the tensile test for *n* = 10 using the test piece.

We examined whether the proposed spring model was applicable to the kirigami structure by comparing the measured values when *n* was changed using Equations (4) and (5). Figure 4 shows the stress–strain diagram using the tensile test. When *n* was 25, the stroke reached the upper limit of the electric measurement device, and the test piece did not break. The elongation stress per strain and breaking strain were calculated using the stress–strain diagram in Figure 4. Figure 5 shows the relationship between the mechanical characteristics of the kirigami structure and *n*. As shown in Figure 5a, elongation stress per strain decreased as *n* increased. As shown in Figure 5b, breaking strain also increased as *n* increased. In the case of *n* = 10 where the influence of the non-uniformed cuts at both ends is large, *E*_c_(*ε*_c_) increased by about 20% and *ε*_cb_ decreased by about 15%, compared to the case of *n* = ∞. The case of *n* = ∞ means the ideal case occurs without the influence of the non-uniformed cuts at both ends. These graphs indicated that the increase in *n* reduced the influence of non-uniform deformation in both end regions, and the uniform deformation in the center region became predominant toward determining the elongation stress per strain and breaking strain of the overall device. The values of *E*_0_(*ε*_0_), *E*_1_(*ε*_1_), *ε*_0b_, and *ε*_1b_ were then calculated from the measured values *E*_c_(*ε*_c_), *ε*_0b_, and *ε*_1b_ on various number of pattern cycles *n* as shown in Figure 5. The fitting curves using the least squares method in Figure 5 were produced using Equations (4) and (5). In Figure 5, we show that the values of the elongation stress per strain are *E*_0_(*ε*_0_) = 32.8 MPa and *E*_1_(*ε*_1_) = 0.878 MPa at *σ*_c_ = 1.0 MPa, *E*_0_(*ε*_0_) = 7.78 MPa and *E*_1_(*ε*_1_) = 1.49 MPa at *σ*_c_ = 2.0 MPa, *E*_0_(*ε*_0_) = 5.83 MPa and *E*_1_(*ε*_1_) = 1.78 MPa at *σ*_c_ = 3.0 MPa. In addition, we found that the values of the breaking strain were *ε*_0b_ = 0.451 strain, and *ε*_1b_ = 1.41 strain. It was found that the proposed spring model was applicable to the kirigami structure, because all the measured values fit the theoretical curves of Equations (4) and (5). We confirmed whether the predicted curve of the stress–strain diagram from the spring model shows the mechanical characteristics of the overall kirigami-structured device. When *n* = 10, the non-uniformly deformed regions at both ends predominantly influence the mechanical characteristics of the overall device. The average values of the elongation stress per strain and breaking strain of the overall device at *n* = 10 in Figure 4 were plotted in the stress–strain diagram in Figure 6. The measured values of *E*_0_(*ε*_0_), *E*_1_(*ε*_1_)_,_ *ε*_0b_, and *ε*_1b_ for 10 pattern cycles were substituted into Equations (4) and (5). Based on these equations, the model values of the elongation stress per strain and breaking strain for *n* = 10 were plotted in the stress–strain diagram in Figure 6. The values derived from the equations and the measured values were compared and found to be in good agreement. Accordingly, theoretical design of the mechanical characteristics of the kirigami structure was made possible by applying the spring model to the kirigami structure; the model can well predict the influence of cuts, which induce non-uniform deformation at both ends.

## 4. Conclusions

In order to design the mechanical characteristics of a kirigami structure theoretically, we modeled the stretching of such a structure when considering the influence of cuts inducing non-uniform deformation at both ends. The stress–strain diagram for the stretched kirigami structure showed non-linear curves. Thus, we considered elongation stress per strain and breaking strain to characterize the kirigami structure mechanically. For the modeling, we focused on the difference in the deformation degree of the cut and divided the kirigami structure into three regions: two non-uniform deformed regions for one cycle at both ends, and one uniform deformed region at the center. We proposed a model of connected springs in series, which had different elongation stress per strain depending on the regions, considering the cuts in one cycle as one spring. We derived formulas from the spring model, which showed the dependence of elongation stress per strain and breaking strain of the kirigami structure on the number of pattern cycles *n*. Comparing the derived formulas and the measured values, we validated the proposed model. The values provided by the formulas of the spring model were in good agreement with the values measured from the tensile test for both elongation stress per strain and breaking strain, illustrating that the proposed spring model was applicable to the kirigami structure. Our proposed spring model well predicted the tendency of the stress–strain diagram curve of the kirigami structure test piece with *n* = 10, and compared to case of *n* = ∞, it showed the large influence of the non-uniform deformation at both ends, *E*_c_(*ε*_c_) increased by about 20% and *ε*_cb_ decreased by about 15%. Based on the above, it is possible to theoretically design the mechanical characteristics of a kirigami structure for certain values of *w*, *d* and *p,* while considering the influence of non-uniformly deformed cuts at both ends. This can be accomplished by calculating *E*_0_(*ε*_0_), *E*_1_(*ε*_1_), *ε*_0b_, and *ε*_1b_ in Equations (4) and (5) by fitting from measured values.
